# Defining a novel subset of CD1d‐dependent type II natural killer T cells using natural killer cell‐associated markers

**DOI:** 10.1111/sji.12794

**Published:** 2019-06-26

**Authors:** Avadhesh Kumar Singh, Sara Rhost, Linda Löfbom, Susanna L. Cardell

**Affiliations:** ^1^ Department of Microbiology and Immunology Institute of Biomedicine, University of Gothenburg Gothenburg Sweden; ^2^ Present address: Department of Laboratory Medicine, Institute of Biomedicine, Sahlgrenska Cancer Center University of Gothenburg Gothenburg Sweden; ^3^ Present address: Fujirebio Diagnostics AB Gothenburg Box 12132 Sweden

**Keywords:** cytokines, experimental animals, natural killer T cells, transcription factors

## Abstract

Natural killer T (NKT) cells are αβ T cell receptor (TCR) expressing innate‐like T cells that display natural killer (NK) cell markers. Based on TCR characteristics, they are divided into two groups restricted to the MHC class I‐like molecule CD1d. Type I NKT cells, most extensively studied, are identified by a semi‐invariant Vα14‐Jα18 (mouse, Vα24‐Jα18 in humans) TCR reactive to the prototypic ligand α‐galactosylceramide presented on CD1d. In contrast, type II NKT cells display diverse TCR reacting to different CD1d‐presented ligands. There are no reagents that identify all type II NKT cells, limiting their exploration. Here, we searched for novel type II NKT cells by comparing Jα18^−/−^MHCII^−/−^ mice that harbour type II but not type I NKT cells, and CD1d^−/−^MHCII^−/−^ mice, lacking all NKT cells. We identified significantly larger populations of CD4^+^ and CD4^−^CD8^−^ (double negative, DN) TCRβ^+^ cells expressing NKG2D or NKG2A/C/E in Jα18^−/−^MHCII^−/−^ mice compared with CD1d^−/−^MHCII^−/−^ mice, suggesting that 30%‐50% of these cells were type II NKT cells. They expressed CD122, NK1.1, CXCR3 and intermediate/low levels of CD45RB. Further, the CD4^+^ subset was CD69^+^, while the DN cells were CD49b^+^ and CD62L^+^. Both subsets expressed the NKT cell‐associated promyelocytic leukaemia zinc finger (PLZF) transcription factor and Tbet, while fewer cells expressed RORγt. NKG2D^+^ CD4^+^ and DN populations were producers of IFN‐γ, but rarely IL‐4 and IL‐17. Taken together, we identify a novel subset of primary CD4^+^ and DN type II NKT cells that expresses NKG2 receptors have typical NKT cell phenotypes and a TH1‐like cytokine production.

## 1 INTRODUCTION

Natural killer T (NKT) cells are innate‐like T lymphocytes that express αβ T cell receptors (TCR) together with natural killer (NK) cell lineage markers (such as NK1.1 or CD49b in the mouse and CD161 in humans) and possess functional properties of both T and NK cells.^1^ In contrast to conventional αβ T cells, NKT cells recognize both exogenous and endogenous lipid antigens presented on CD1d, an MHC class I‐like antigen‐presenting molecule. Based on the nature of their TCR, NKT cells comprise two main subsets, invariant (*i*) or type I NKT cells and diverse or type II NKT cells. Type I NKT cells, which is the most extensively studied subgroup, express a semi‐invariant Vα14‐Jα18 TCR in mice and Vα24‐Jα18 in humans, paired with diverse TCR β‐chains using Vβ8.2, Vβ7 and Vβ2 in mice and Vβ11 in humans.^2^, ^3^, ^4^ The less explored type II NKT cells utilize a diverse TCR repertoire both in mice and humans.^5^, ^6^, ^7^, ^8^

Type I NKT cells are reactive to the glycolipid α‐galactosylceramide (α‐GalCer), a strong inducer of anti‐tumour immunity in mice.^9^, ^10^, ^11^, ^12^ When loaded on CD1d‐tetramers, CD1d/α‐GalCer forms a stable reagent that can be used for detection of type I NKT cells.^13^, ^14^ This has been instrumental for generating detailed information on type I NKT cells and has helped to establish an immunoregulatory role of these cells. In contrast, type II NKT cells recognize a diverse repertoire of lipid antigens, and as for today, no unique cell surface marker has been identified for this subset, making broad studies of type II NKT cells difficult.^8^ Significant information has been provided by studies of a transgenic mouse expressing a type II NKT cell TCR.^15^, ^16^, ^17^, ^18^, ^19^ These TCR‐transgenic type II NKT cells (24αβ NKT cells) share several surface markers with type I NKT cells, such as NK1.1, CD122 and intermediate TCR levels, and are either CD4^+^ or CD4^−^CD8^−^.^15^ Further, type II NKT cells express the transcription factor PLZF, required for the development of type I NKT cells.^20^, ^21^, ^22^ Although transgenic 24αβ type II NKT cells share many features with type I NKT cells, 24αβ type II NKT cells exhibit other phenotypic traits (CD62L^hi^, CD69^neg/lo^ and CD49b^+^), effector molecules (production of IFN‐γ rather than IL‐4), NK receptors, integrins and chemokine receptors distinct from type I NKT cells.^8^, ^15^, ^16^, ^17^, ^19^ This suggested that at least some type II NKT cells might preferentially be TH1‐like and have a naive/resting phenotype, while type I NKT cells produce a broader cytokine array and have an activated memory‐like phenotype.

Studies of the TCR reactivity of CD1d‐reactive T cell hybridomas identified sulphatide as a potent ligand for type II NKT cells.^23^, ^24^ Using sulphatide‐loaded CD1d‐tetramers, a population of immunoregulatory sulphatide‐reactive CD1d‐restricted T cells was demonstrated.^23^ These cells suppressed experimental autoimmune encephalomyelitis, had an oligoclonal TCR repertoire and shared characteristics of 24αβ type II NKT cells, being NK1.1^+^ and CD69^−^.^23^, ^25^ Thus, taken together, these studies suggested that type II NKT cells possess antigen specificities, phenotypes and functional properties distinct from those of type I NKT cells.

While some studies have described type II NKT cells with specific TCR/ligand reactivities, another approach to define a broader set of primary type II NKT cells applied 4get mice that express an IL‐4‐eGFP reporter gene. This transgenic reporter marked essentially all type I NKT cells that had previously been shown to contain IL‐4 mRNA at steady state.^26^ Further, making the assumption that also type II NKT cells express steady state IL‐4 mRNA, a CD1d‐dependent, Jα18‐independent, type II NKT cell population was demonstrated in 4get mice.^27^, ^28^ These 4get type II NKT cells were NK1.1^+^ CD69^+^ and CD62L^−^ and had an IL‐4 production similar to type I NKT cells. Thus, type II NKT cells clearly also include cells that are functionally and phenotypically similar to type I NKT cells.

However, considering that TCR‐transgenic and sulphatide‐reactive type II NKT cells display some phenotypic characteristics different from those of the classical type I NKT cells, and may not produce high levels of IL‐4, we postulated that there would be additional primary type II NKT cells not visualized in the 4get model. Here, we have asked whether we can identify primary type II NKT cells with a phenotype similar to previously described transgenic 24αβ type II NKT cells and sulphatide‐reactive type II NKT cells, that is, a predominantly resting and NK‐like phenotype. To this end, we searched for CD1d‐dependent and Jα18‐independent TCRβ^+^ CD4^+^ and CD4^−^CD8^−^ (double negative, DN) cells with these characteristics, exploring two different transgenic mouse lines: Jα18^−/−^MHCII^−/−^ double knockout (DKO) mice lacking type I NKT cells and CD1d^−/−^MHCII^−/− ^DKO mice lacking all CD1d‐dependent NKT cells.

## 2 MATERIALS AND METHODS

### 2.1 Mice

Jα18^−/−^ 29 and CD1d^−/−^ 30 mice were crossed with MHCII^−/−^ mice^31^ to obtain Jα18^−/−^MHCII^−/−^ and CD1d^−/−^MHCII^−/− ^DKO mice, respectively. C57BL/6 (B6) wild‐type (WT) mice were used as controls. All mice were bred and maintained on a C57BL/6 genetic background at the Department of Experimental Biomedicine, University of Gothenburg. Both male and female sex‐ and age‐matched mice 12‐16 weeks of age were used for experiments. The studies were approved by the Animal Research Ethics Committee of Gothenburg, and regulatory animal experimentation guidelines were followed.

### 2.2 Cell preparation and flow cytometry

Single‐cell suspensions were prepared from spleens and used for flow cytometry staining. CD1d/PBS57 tetramers were kindly provided by the NIH tetramer core facility. Briefly, cells were suspended in FACS buffer (PBS containing 0.02% NaN_3_ and 2% FBS), Fc‐blocked with anti‐mouse 2.4G2 antibody, and subsequently, for the WT B6 mice, cells were stained with brilliant violet (BV) 421‐conjugated CD1d/PBS57 tetramers at room temperature (RT) for 30 minutes. Cells were then stained with anti‐mouse fluorochrome‐conjugated monoclonal antibodies (mAbs) for 30 minutes at 4°C. Following mAbs were used: PE‐Cy7‐B220 (clone RA3‐6B2, eBioscience), PerCp‐Cy5.5‐CD19 (clone 6D5, BioLegend) FITC‐TCRβ (clone H57‐597, eBioscience), Alexa Fluor 700 or PerCp‐CD4 (clone RMA4‐4 or RMA4‐5, BioLegend), Pacific Blue or APC‐CD8 (clone 53‐6.7, BioLegend), PE‐CD44 (clone IM7, eBioscience), PE‐CD69 (clone H1.2F3, BioLegend), PE‐CD62L (clone MEL‐14, eBioscience), PE‐CD122 (clone 5H4, BioLegend), PE‐CD49b (clone DX5, eBioscience), APC‐Ly49G2 (clone 4D11, BD Biosciences, San Jose, CA, USA), PE‐NKG2A/C/E (reactive to NKG2A, NKG2C and NKG2E), PE or APC‐NKG2D (clone CX5, BioLegend), PE‐PD1 (clone J43, eBioscience), PE or APC‐NK1.1 (clone PK136, eBioscience), PE‐CD45RB (clone C363‐16A, BioLegend) and PE‐CXCR3 (clone CXCR3‐173, eBioscience).

### 2.3 Stimulation and staining for intracellular cytokines

For intracellular staining, B220/CD11b/CD11c/CD8α‐positive splenocytes from the DKO mice were first depleted by magnetic‐activated cell sorting (MACS) using anti‐mouse biotinylated mAbs against B220 (clone RA3‐6B2, BioLegend), CD11b (Clone M1/70, BioLegend), CD11c (clone HL3, BD Biosciences) and CD8α (clone 53‐6.7, BD Biosciences), and anti‐biotin MicroBeads (Miltenyi Biotec, Bergisch Gladbach, Germany) following manufacturer's instructions. Enriched cells were stimulated with PMA (50 ng/mL) and ionomycin (500 ng/mL; Sigma Aldrich) in the presence of Brefeldin A (eBioscience) in RPMI complete medium (10% foetal bovine serum, 1% penicillin‐streptomycin, 1% HEPES, 1% NaP and 0.1% β‐mercaptoethanol) for 4 hours in 5% CO_2 _at 37°C. Cells were surface stained as described above, fixed and permeabilized using intracellular fixation and permeabilization buffer (eBioscience) and stained with PE‐IFN‐γ (clone XMG1.2), PE‐IL‐4 (clone 11B11) and PE‐IL‐17 (clone TC11‐18H10) mAbs (all from BD Biosciences). Transcription factors in unstimulated cells were stained with PE‐promyelocytic leukaemia zinc finger (PLZF; clone Mags.21F7, eBioscience), PE‐Cy7‐Tbet (clone 4B10, eBioscience) and PE‐CF594‐RORγt (clone Q31‐378, BD Biosciences) mAbs using Foxp3 staining buffer (eBioscience). Cells were always stained with LIVE/DEAD® aqua stain (Life Technologies), and fluorescence minus one (FMO) was used as background control. Data were acquired using LSRII (BD Biosciences) cytometer and analysed using FlowJo v.10 software (Tree Star, Inc).

### 2.4 Statistical analyses

The unpaired two‐tailed Mann‐Whitney *U* test evaluated statistical differences between groups. Data are expressed as mean ± SD for each group, and *P*‐values < 0.05 were considered to be statistically significant. These tests were performed using Prism GraphPad Software, version 8.

## 3 RESULTS

### 3.1 Both Jα18^−/−^MHCII^−/−^ and CD1d^−/−^MHCII^−/−^ mice have significant populations of splenic CD4^+^ and DN TCRβ^+^ cells

**Figure 1:**
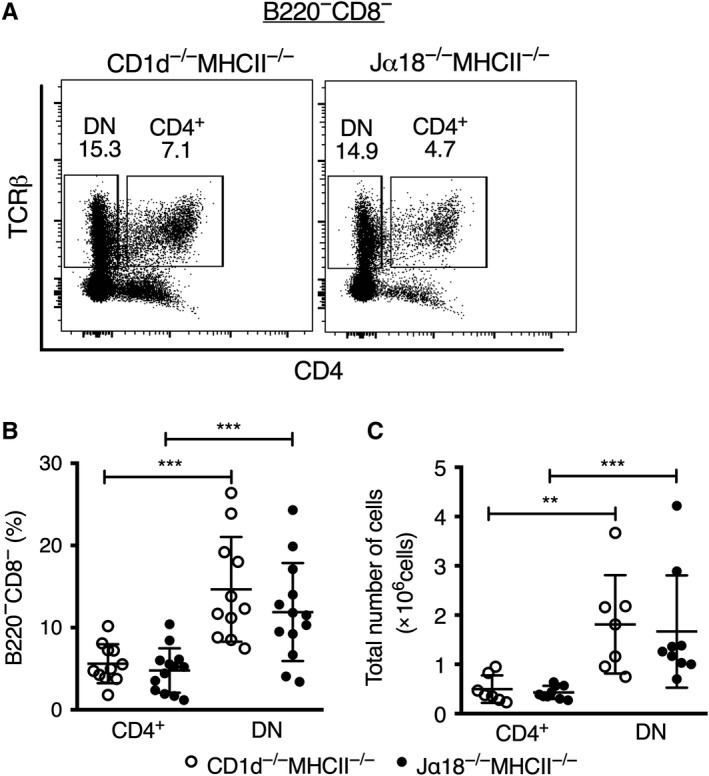
CD4^+^ and DN TCRβ^+^ splenocyte frequencies in DKO mice. Spleen cells were prepared and stained for different surface markers. A, Representative flow cytometry plots showing gatings for CD4^+^ and DN splenic TCRβ^+^ cells from CD1d^−/−^MHCII^−/−^ and Jα18^−/−^MHCII^−/−^ mice. B, C, Scatter plots illustrate frequencies among B220^−^CD8^−^ cells (left) and absolute numbers per spleen (right) of CD4^+^ and DN TCRβ^+^ cells. Results are expressed as mean ± SD; each dot represents values from one mouse. ** indicates *P* < 0.001 and *** *P* < 0.0001 as calculated by Mann‐Whitney *U* test (two‐tailed)

To search for a CD1d‐dependent Jα18‐independent T cell population, total CD4^+^ and CD4^−^CD8^−^ DN TCRαβ^+^ T cells were quantified in spleens of Jα18^−/−^MHCII^−/−^ and CD1d^−/−^MHCII^−/−^ DKO mice (Figure 1). Both DKO lines are deficient in conventional CD4 T cells and were used to facilitate identification and investigation of type II NKT cells present in Jα18^−/−^MHCII^−/− ^DKO mice. Flow cytometry analysis revealed significant populations of both CD4^+^ and DN TCRαβ^+^ T cells in the two mouse lines. The DN subset was 3‐ to 4‐fold larger than the CD4^+^ population in both mouse lines; however, there were no significant differences in numbers or frequencies of CD4^+^ or DN TCRαβ^+^ T cells between Jα18^−/−^MHCII^−/−^ and CD1d^−/−^MHCII^−/−^ DKO mice (Figure 1).

### 3.2 NKT‐like cells are present among splenic CD4^+^ and DN TCRβ^+^ cells in DKO mice

**Figure 2:**
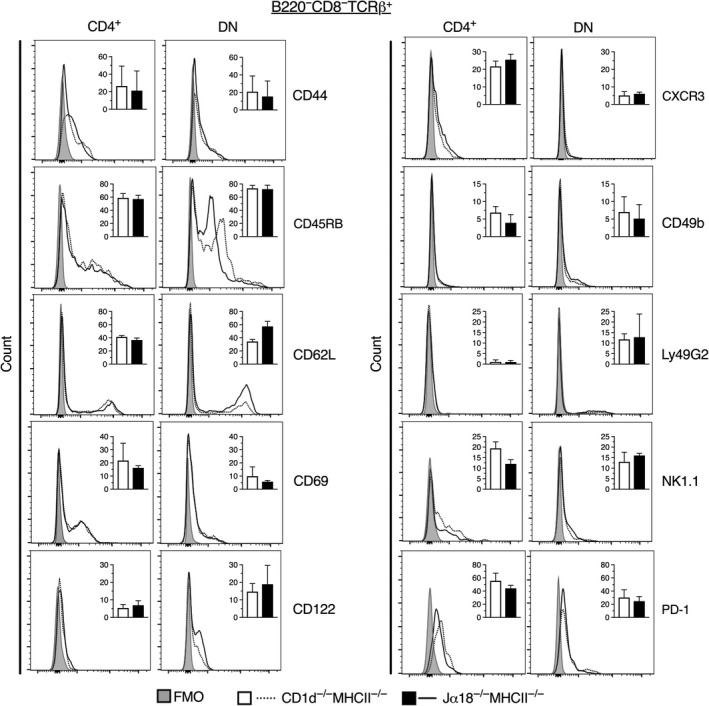
Expression level of surface markers on CD4^+^ and DN TCRβ^+^ splenocytes from DKO mice. Spleen cells were prepared and stained for different surface markers. Representative flow cytometric histograms display the expression of indicated markers on CD4^+^ and DN TCRβ^+^ cells. Dotted lines represent CD1d^−/−^MHCII^−/− ^mice, and solid lines represent Jα18^−/−^MHCII^−/−^ mice. Shaded area shows FMO for background fluorescence. Inlet bar diagrams within the respective histograms are summaries of either three (CD44, CD45RB, CD62L, CXCR3, Ly49G2, NK1.1 and PD‐1) or four independent experiments (CD69, CD122 and CD49b), with a total of 6 and 8 mice, respectively, and expressed as mean frequencies of positive cells ± SD. White bars represent CD1d^−/−^MHCII^−/− ^mice, and black bars Jα18^−/−^MHCII^−/−^ mice. Statistics were analysed using Mann‐Whitney *U* test (two‐tailed)

**Figure 3:**
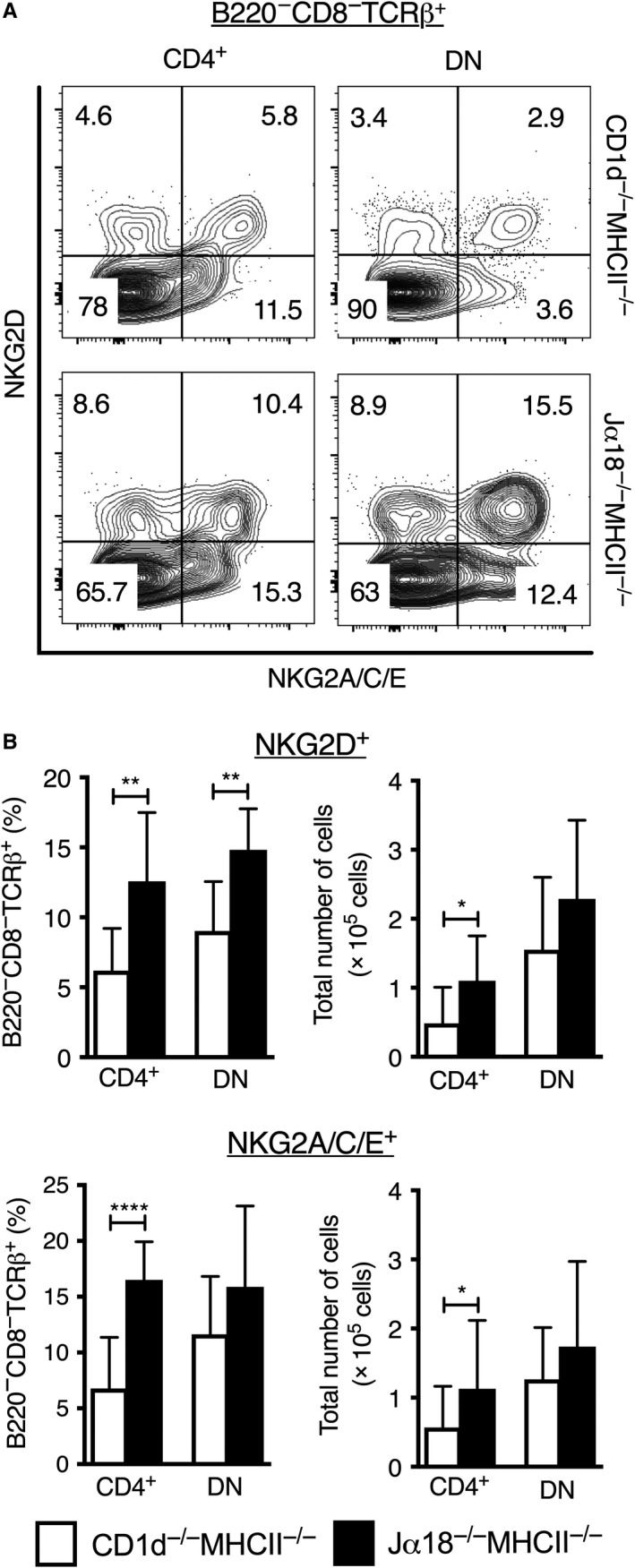
Increased expression of NKG2 receptors on CD4^+^ and DN splenic TCRβ^+^ cells in Jα18^−/−^MHCII^−/−^ compared to CD1d^−/−^MHCII^−/−^ mice. Spleen cells were prepared and stained for different surface markers. A, Flow cytometric plots display the expression patterns of NKG2D and NKG2A/C/E on CD4^+^ and DN splenic TCRβ^+^ cells from CD1d^−/−^MHCII^−/−^ and Jα18^−/−^MHCII^−/−^ mice. B, Frequencies among B220^−^CD8^−^TCRβ^+^ splenocytes gated as in (A) (left) and absolute numbers (right) of NKG2D and NKG2A/C/E expressing CD4^+^ and DN TCRβ^+^ cells. White bars represent CD1d^−/−^MHCII^−/−^, and black bars represent Jα18^−/−^MHCII^−/−^ DKO mice. Results are expressed as mean ± SD of 8 mice from four independent experiments; * indicates *P* < 0.001, ** *P* < 0.001 and **** *P* < 0.00001 as calculated by Mann‐Whitney *U* test (two‐tailed). The flow cytometry plots of co‐expression of NKG2D and NKG2A/C/E on CD4^+^ and DN T cells in DKO strains shown in (A) is representative of two experiments

Next, we investigated the expression of different surface markers, including NK‐associated and other markers expressed differentially on NKT subsets, within the CD4^+^ or DN TCRαβ^+^ T cell subsets in both DKO mouse lines (Figure 2 and Figure 3). We included a set of markers that distinguish naive and memory T cells (CD44, CD62L and CD45RB) and markers generally expressed by NKT/NK cells (NK1.1, CD69, CD122, Ly49G2, CXCR3 and CD49b; Figure 2). None of these markers differed significantly in expression between Jα18^−/−^MHCII^−/−^ and CD1d^−/−^MHCII^−/−^ CD4^+^ or DN T cell subsets. Both CD4^+^ and DN populations demonstrated some expression of CD44, a marker of effector‐memory T cells that is also found on NKT cells, and they were intermediate/low for CD45RB. CD62L was expressed on a fraction of both subsets, with a tendency of higher expression on the DN subset from Jα18^−/−^MHCII^−/−^ mice compared with the same subset in CD1d^−/−^MHCII^−/−^ mice. This suggested that both subsets predominantly consisted of effector‐/memory‐like cells (CD44^+^, CD62L^–^) in the two mouse strains. Further, comparing CD4^+^ and DN cells, the expression of CD69, CXCR3 and PD‐1 was higher on the CD4 subsets, while CD122 and Ly49G2 expression was higher on DN cells. Taken together, these results demonstrate that both CD4^+^ and DN TCRβ^+^ cells from the DKO mice contain populations of cells similar to NKT cells, with a memory‐like phenotype and expression of NK markers.

### 3.3 Splenic CD4^+^ and DN TCRβ^+^ cells expressing NKG2A/C/E or NKG2D are more frequent in Jα18^−/−^MHCII^−/−^ mice compared with CD1d^−/−^MHCII^−/−^ mice

The expression of the NK/NKT cell‐associated NKG2 receptors was also investigated (by the use of an antibody reactive to NKG2D, and a separate antibody simultaneously reactive to the multiple isoforms NKG2A, NKG2C and NKG2E, Figure 3). Frequencies of NKG2D expressing cells among both CD4^+^ and DN T cells were significantly higher in Jα18^−/−^MHCII^−/−^ mice compared with CD1d^−/−^MHCII^−/−^ DKO mice (Figure 3). Similarly, the percentage of NKG2A/C/E^+^ cells was also significantly elevated among CD4^+^ cells in Jα18^−/−^MHCII^−/−^ mice. Further, the absolute numbers of CD4^+^ T cells expressing NKG2A/C/E or NKG2D were a significantly higher in Jα18^−/−^MHCII^−/− ^mice (Figure 3B). Moreover, splenocytes from Jα18^−/−^MHCII^−/− ^mice had higher frequencies of CD4^+^ and DN T cells co‐expressing NKG2A/C/E and NKG2D compared with CD1d^−/−^MHCII^−/−^ mice (Figure 3A). This demonstrates an enrichment of NKG2D‐ and NKG2A/C/E expressing CD4^+^ and DN T cells in Jα18^−/−^MHCII^−/−^ mice compared with CD1d^−/−^MHCII^−/−^ mice and suggests that around half of the CD4^+^ and DN cells expressing NKG2D or NKG2A/C/E in Jα18^−/−^MHCII^−/−^ mice may be CD1d‐restricted type II NKT cells. Considering the degree of co‐expression of NKG2D and NKG2A/C/E, CD1d‐restricted type II NKT cells within these populations would make up an estimate of around 10% of all CD4^+^ and DN T cells in Jα18^−/−^MHCII^−/− ^mice.

### 3.4 Splenic NKG2D^+^ CD4^+^ and DN TCRβ^+^ cells are enriched for cells expressing activation/memory and NKT cell‐associated markers

**Figure 4:**
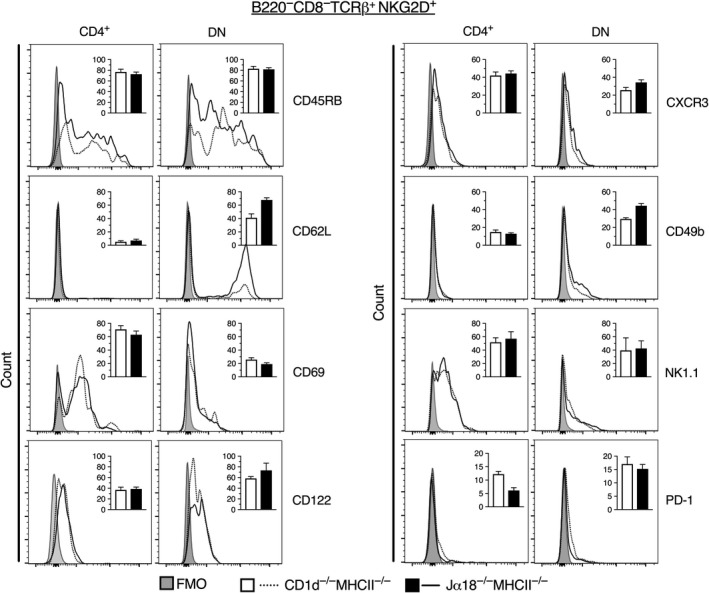
Expression of surface markers on NKG2D^+^ CD4^+^ and DN TCRβ^+^ splenocytes from DKO mice. Spleen cells were prepared and stained for different surface markers. Representative flow cytometric histograms display the expression of indicated markers on NKG2D^+^ CD4^+^ and NKG2D^+^ DN TCRβ^+^ cells. Dotted lines represent CD1d^−/−^MHCII^−/−^, and solid lines represent Jα18^−/−^MHCII^−/−^ mice. Shaded area shows the FMO for background fluorescence. Inlet bar diagrams within the respective histograms are summaries of 6 mice from three independent experiments, and are expressed as mean frequencies of positive cells ± SD. White bars represent CD1d^−/−^MHCII^−/− ^mice, and black bars Jα18^−/−^MHCII^−/−^ mice. Statistics were analysed using Mann‐Whitney *U* test (two‐tailed)

To determine whether NKG2D^+^ CD4^+^ and DN T cells had the previously described phenotype of type II NKT cells, we investigated these populations for the expression of some of the initially investigated cell surface markers (Figure 4). Both populations expressed intermediate/low levels of CD45RB. Compared with the total CD4^+^ and DN populations, cells expressing CD122, CXCR3 and NK1.1 were enriched among both CD4^+^ and DN NKG2D^+^ cells (Figure 2 vs Figure 4). Further, while CD69 was highly enriched among CD4^+^ NKG2D^+^ cells, CD62L expression was strongly reduced. CD49b was instead enriched among DN NKG2D^+^ cells that remained CD62L high and CD69 low. Interestingly, PD‐1 expression was lower on both NKG2D^+^ populations compared with total CD4^+^ and DN cells. Taken together, our data show that both CD4^+^ and DN NKG2D^+^ splenic T cells were enriched for surface markers associated with NKT cells. The CD4 subset had markers of pre‐activated/memory T cells, and the DN subset expressed NK‐associated markers. Furthermore, there was a clear tendency of higher expression of CD62L, CD122 and CD49b on DN NKG2D^+^ cells from Jα18^−/−^MHCII^−/−^ compared with CD1d^−/−^MHCII^−/−^ mice, which is similar to the phenotype described for both the TCR‐transgenic and sulphatide‐reactive type II NKT cells; however, the differences did not reach statistical significance.

### 3.5 PLZF is expressed in TCRβ^+^ NKG2D^+^ CD4^+^ and DN cells in DKO mice

**Figure 5:**
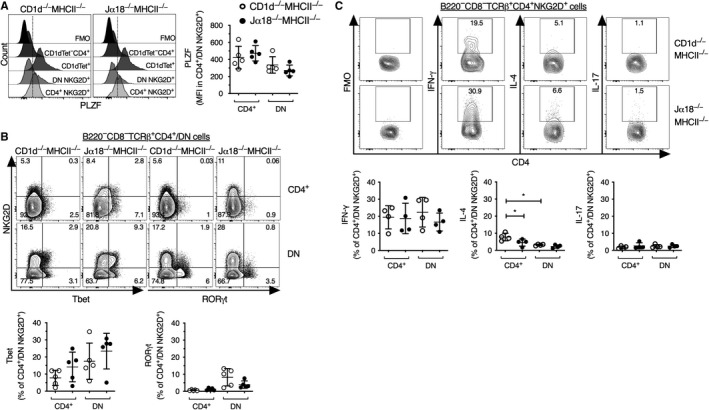
Transcription factor and cytokine expression in NKG2D^+^ CD4^+^ and DN TCRβ^+^ cells. Splenocytes were isolated and depleted of cells positive for B220, CD11b, CD11c and CD8α and stained for surface markers and intracellular transcription factors (A, B). A, Representative flow cytometric histograms displaying the expression of PLZF in the indicated cell types from CD1d^−/−^MHCII^−/− ^and Jα18^−/−^MHCII^−/−^ mice (left). α‐GalCer loaded CD1d‐tetramer^+^ CD4^+^ cells (CD1dTet^+^) and CD4^+^ cells negative for α‐GalCer/CD1d‐tetramer staining (CD1dTet^‐^CD4^+^) from wild‐type C57BL/6 mice are shown in each overlay as reference. The dotted line indicates gate for PLZF positive staining. Scatter plots for median fluorescence intensity (MFI) of PLZF expression within the indicated cells are shown (right). B, Representative flow cytometric contour plots showing the expression of Tbet and RORγt within the indicated cell types from CD1d^−/−^MHCII^−/− ^and Jα18^−/−^MHCII^−/−^ mice (upper panel). Scatter plots for the expression of Tbet and RORγt are shown (lower panel). C, Splenocytes depleted of cells positive for B220, CD11b, CD11c and CD8α were stimulated with PMA and ionomycin and thereafter stained for surface markers and intracellular cytokines. Representative flow cytometric contour plots showing the expression of indicated cytokines and background FMO (fluorescence minus one) within B220^−^CD8^−^TCRβ^+^CD4^+^NKG2D^+^ cells (upper panel, see also Supportive data Figure S1C). Scatter plots show IFN‐γ, IL‐4 and IL‐17 production by NKG2D^+^ CD4^+^ and NKG2D^+^ DN TCRβ^+^ cells from CD1d^−/−^MHCII^−/− ^and Jα18^−/−^MHCII^−/−^ DKO mice (lower panel). Results are expressed as mean ± SD. Each dot represents one experiment with splenocytes pooled from two mice. * indicates *P* < 0.01 as calculated by Mann‐Whitney *U* test (two‐tailed) used to assess statistical significance

Unlike conventional T cells, type I NKT cells depend on the transcription factor PLZF for their development.^20^, ^21^ Studies using TCR‐transgenic mice and the 4get model suggest that also type II NKT cells depend on PLZF.^27^, ^28^ Therefore, we investigated the expression of PLZF in NKG2D positive CD4^+^ and DN TCRβ^+^ cells from the DKO mice (Figure 5A). As shown before, CD4^+^ (and DN, data not shown) type I NKT cells from WT mice demonstrated PLZF expression, while conventional CD4^+^ T cells were negative for this transcription factor. Total CD4^+^ TCRβ^+^ T cells in DKO mice contained 5%‐30% of PLZF^hi^ cells; however, essentially all NKG2D^+ ^CD4^+^ cells expressed PLZF at intermediate levels (Figure 5A and Figure S1A). PLZF^+^cells were also enriched (on average 7%‐15%) among NKG2D^+ ^DN cells compared with total DN cells (on average 6%‐11%; Figure 5A, Figure S1A). Staining of PLZF in NKG2A/C/E^+^ CD4^+^ and DN cells yielded similar results (Figure S1B). This shows that CD4^+^ and DN NKG2D^+ ^cells in both DKO strains display significant expression of the NKT cell‐associated transcription factor PLZF.

### 3.6 CD4^+^NKG2D^+ ^cells from DKO mice produce IFN‐γ but little IL‐4 and IL‐17

Previously, functional subsets of type I NKT cells have been defined that produce different sets of cytokines similar to the conventional CD4^+^ cells denoted TH1, TH2 and TH17 cells.^32^ As for conventional CD4^+^ cells, the cytokine production by these type I NKT cell subsets is associated with the expression of different transcription factors.^32^ Type I NKT cells that express Tbet and intermediate levels of PLZF are mainly producers of IFN‐γ are termed NKT1 cells. In contrast, NKT2 cells produce IL‐4 and have high levels of PLZF and low levels of Tbet, while NKT17 cells express PLZF and RORγt and produce IL‐17. Figure 5B shows that the NKG2D^+^ DKO T cell population contained Tbet positive cells, generally more frequent in the DN (around 5%‐30% positive) than in the CD4^+^ (around 2%‐25%) subset (Figure 5B). Compared with Tbet, RORγt was expressed by lower frequencies of NKG2D^+^ DKO T cells (around 1% or less of CD4^+^ cells, and 2%‐15% of DN cells; Figure 5B). However, the expression of PLZF, Tbet or RORγt in TCRβ^+^ CD4^+^ and DN NKG2D^+^ cells did not differ between DKO strains. Taken together, the data show that the CD4^+^ and DN cells expressing NKG2D or NKG2A/C/E in Jα18^−/−^MHCII^−/− ^mice and CD1d^−/−^MHCII^−/−^ mice expressed PLZF and Tbet, but RORγt^+^ cells were rare.

To investigate whether the transcription factor expression reflected the capacity to produce cytokines, we stimulated cells in vitro with PMA and ionomycin. Upon activation, on average around 20% of the CD4^+^ and DN NKG2D^+^ T cells produced IFN‐γ, while lower frequencies produced IL‐4 or IL‐17 (Figure 5C and Figure S1C). Comparing populations from the two DKO mouse strains, we found that IFN‐γ‐ and IL‐17‐producing cells were similar in frequency in these mice. In contrast, the proportion of IL‐4‐producing CD4^+^NKG2D^+^ cells in Jα18^−/−^MHCII^−/− ^mice was lower compared with those from CD1d^−/−^MHCII^−/− ^mice. In addition, IL‐4‐production was higher in CD4^+^NKG2D^+^ cells than in DN NKG2D^+^ cells in CD1d^−/−^MHCII^−/− ^mice. Thus, the cytokine profile was indeed consistent with the expression of transcription factors and suggests that CD4^+^ and DN NKG2D^+^ cells from both DKO mouse strains are predominantly NKT1 like, expressing PLZF and Tbet and producing IFN‐γ after stimulation.

## 4 DISCUSSION

Here, we aimed to identify and characterize primary type II NKT cells with an NK/TH1‐like phenotype, to broaden our knowledge on type II NKT cells.

We selected a number of markers associated with NK/NKT cells and screened CD4^+^ and DN TCRβ^+^ T cells looking for populations expanded in Jα18^−/−^MHCII^−/− ^mice compared with CD1d^−/−^MHCII^−/− ^DKO mice. We identified a subset of CD1d‐dependent type II NKT cells among NKG2D and NKG2A/C/E expressing cells. NKG2 molecules are type II transmembrane receptors encoded in the NK gene complex. NKG2A is an inhibitory receptor, while NKG2C, E and NKG2D are activating receptors. Ligands for these receptors are upregulated upon stress or inflammation, and on transformed cells. Like several other NK markers, the NKG2 molecules are found on NKT cells. A significant proportion of CD4^+^ and DN T cells in both DKO mice co‐expressed NKG2A/C/E and NKG2D. Further phenotypic analysis showed that both CD4^+^ and DN NKG2D^+^ T cells were enriched for expression of NK1.1, CD122 and CXCR3, all markers associated with NKT cells.

CD4^+^ NKG2D^+^ T cells in DKO mice had a phenotype of memory T cells being CD69^+^, negative for CD62L and with low expression of CD45RB, reminiscent of type I NKT cells. In contrast, DN NKG2D^+^ T cells from DKO mice had a relatively naive phenotype as they expressed low levels of CD69 and were CD62L positive. Further, there was an enrichment of cells positive for CD62L and CD49b among DN NKG2D^+^ T cells from Jα18^−/−^MHCII^−/− ^mice compared with CD1d^−/−^MHCII^−/− ^mice, suggesting that DN CD1d‐dependent type II NKT cells were CD62L^+^ CD49b^+^. CD49b is found on all NK cells and on NKT cells, and has been used as an alternative marker on NK/NKT cells in NK1.1‐negative mouse strains. We have previously shown that CD49b is found at higher levels on TCR‐transgenic type II NKT cells than on TCR‐transgenic type I NKT cells, consistent with the phenotype of the DN type II NKT cells found here. Further, the transgenic type II NKT cells were CD62L^+^ CD69^–^,^15^, ^16^, ^33^ similar to the NK1.1^+^ CD69^–^ phenotype of sulphatide/CDd‐tetramer^+^ type II NKT cells.^25^ The NKG2D^+^ CD4^+^ and DN T cells produced IFN‐γ and very little IL‐4 which is distinct from the Vα14‐Jα18 type I NKT cells, that can produce large amounts of cytokines including IL‐4, upon activation.^1^ The predominant IFN‐γ production is coherent with the significant expression of Tbet in the NKG2D^+^ CD4^+^ and DN T cells.

The NKT cell transcription factor PLZF was found in all CD4^+^ cells and a significant proportion of DN T cells positive for NKG2D or NKG2A/C/E from Jα18^−/−^MHCII^−/− ^mice, consistent with the presence of NKT cells within these populations. The PLZF expression was very similar to what has been shown before for TCR‐transgenic 24αβ type II NKT cells (22 and our unpublished data). The fact that all CD4^+^ T cells displaying NKG2A/C/E or ‐D expressed PLZF, although not all of these cells were CD1d‐dependent (as PLZF was seen in these populations also in CD1d^−/−^MHCII^−/−^ mice), demonstrates that other non‐conventional T cells with similar phenotype are present in this population. This is not unexpected, as both CD1d‐dependent and CD1d‐independent NK1.1^+^ TCRβ^+^ cells exist in C57BL/6 mice.^34^, ^35^ Other unconventional T cells such as mucosal‐associated invariant T (MAIT) cells and subsets or TCRγδ T cells also express PLZF,^21^, ^36^, ^37^, ^38^ and there may be other less well described unconventional T cell with a similar phenotype.

The first efforts to characterize primary type II NKT cells were performed using MHC class II‐deficient mice.^39^, ^40^ More recently, using 4get mice, significantly larger populations of GFP‐positive T cells were identified in different lymphoid organs of Jα18‐deficient 4get mice compared with CD1d^−/−^ 4get mice, demonstrating GFP‐marked CD1d‐restricted type II NKT cells in 4get mice.^27^, ^28^ However, type II NKT cells that lack constitutive production of IL‐4 would escape detection in this model. The 4get type II NKT cells had somewhat reduced IFN‐γ production compared with type I NKT cells, but had relatively high IL‐4 production. Further, they provided a rapid cytokine response in vivo after injection of anti‐CD3 antibody, a hallmark of type I NKT cells. In contrast, among NKG2D^+^ type II NKT cells IFN‐γ producers were several fold more frequent than IL‐4 producers. This is consistent with the notion that this TH1‐like subset of type II NKT cells would not be detected in the 4get model. Liver type II NKT cells in 4get mice (GFP^+^ cells in Jα18^−/−^ 4get mice) were phenotypically very similar to type I NKT cells, that is preferentially CD4^+^ but also DN, NK1.1^+^, CD122^+^, CD44^hi^, CD69 ^hi^, CD62L^lo^ and CD45RB^int^.^27^, ^28^ Like type I NKT cells, the 4get type II NKT cells were dependent on PLZF and the adaptor molecule signalling lymphocyte activation molecule‐associated protein (SAP) for their development. Interestingly, the 4get type II NKT cells were activated in CD1d‐dependent manner by the lipid agonist β‐glucosylceramide and lysophosphatidylethanolamine, but not other phospholipids or sulphatide, the latter being a known type II NKT cell lipid antigen. This further supports that some type II NKT cells are not identified in the Jα18^−/−^ 4get model.

In conclusion, both CD4^+^‐ and DN CD1d‐dependent cells expressing the NK cell family receptors NKG2D and NKG2A/C/E were enriched in Jα18^−/−^MHCII^−/− ^mice compared with CD1d^−/−^MHCII^−/− ^mice suggesting that these populations contained significant proportions of type II NKT cells. These cells expressed typical NKT cell markers, were positive for PLZF and Tbet, and produced IFN‐γ but very little IL‐4. The results add to the limited knowledge of primary type II NKT cells by describing CD1d‐dependent type II NKT cells with NK markers and a predominant Th1 cytokine profile.

## CONFLICT OF INTEREST

The authors declare no conflict of interest.

## AUTHOR CONTRIBUTIONS

SC conceived and designed the study, analysed data and wrote the manuscript. AKS and SR performed experiments, analysed data, prepared figures and wrote the manuscript. LL contributed to experiments and reviewed the manuscript.
