# Supplementary figures and images for: Defining a novel subset of CD1d‐dependent type II natural killer T cells using natural killer cell‐associated markers
Source: Scand J Immunol. 2019 Jun 26;90(3):e12794. doi: 10.1111/sji.12794 (PMC6851763; doi:10.1111/sji.12794)

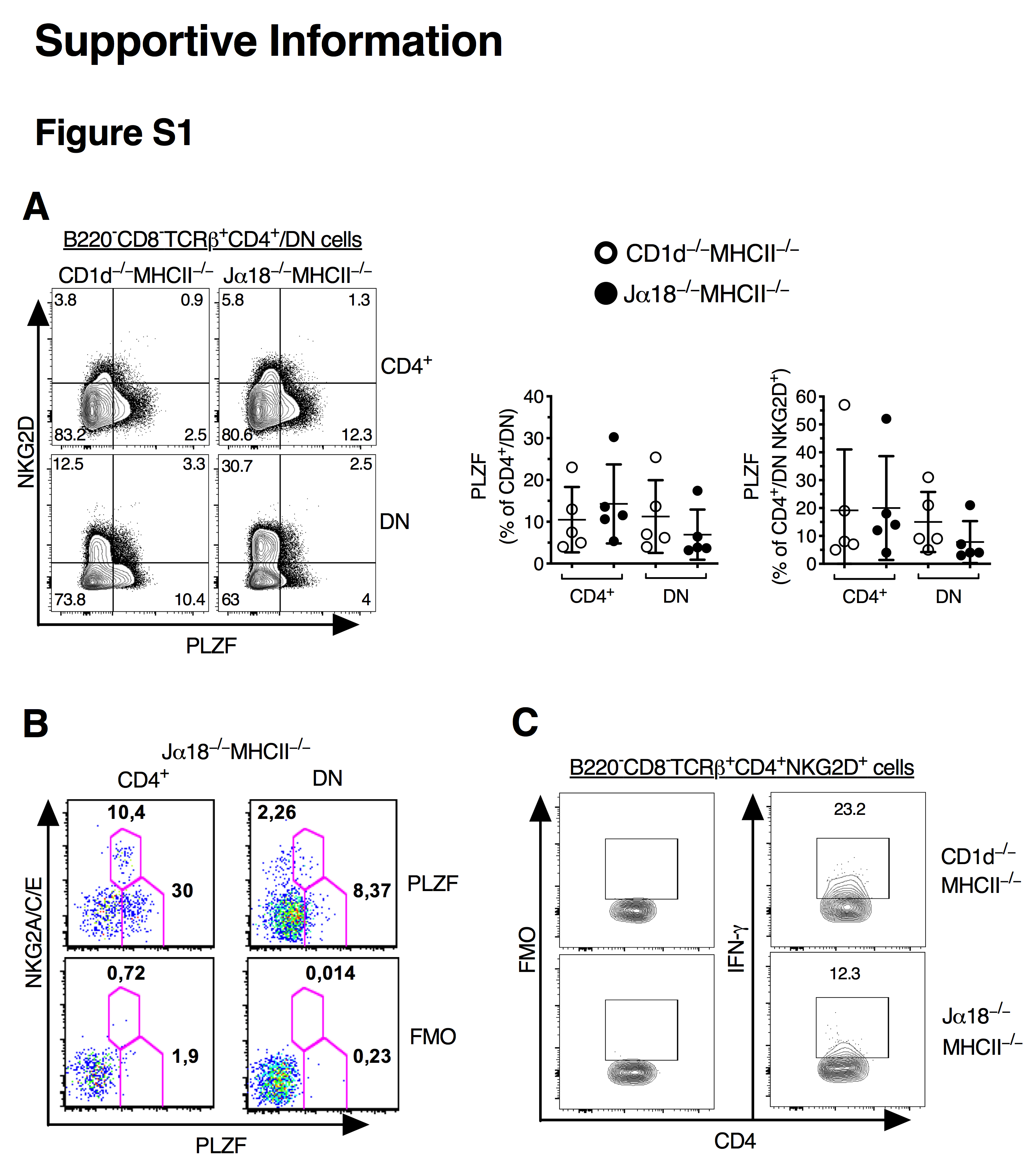

Supplement: Supplementary file 1 [file SJI-90-na-s001.tif]
