# Supplementary material for: Defining a novel subset of CD1d‐dependent type II natural killer T cells using natural killer cell‐associated markers
Source: Scand J Immunol. 2019 Jun 26;90(3):e12794. doi: 10.1111/sji.12794 (PMC6851763; doi:10.1111/sji.12794)
Supplement: Supplementary file 2 [file SJI-90-na-s002.docx]

**Supportive Information**

**Figure S1:** **Transcription factor and cytokine expression in NKG2D^+^ CD4^+^ and DN TCRβ^+^ cells.** Splenocytes were isolated and depleted of cells positive for B220, CD11b, CD11c and CD8α and stained for surface markers and intracellular transcription factors **(A, B)**. **(A)** Representative flow cytometric contour plots displaying the expression of PLZF in the indicated cell types from CD1d^−/−^MHCII^−/−^ and Jα18^−/−^MHCII^−/−^ mice (left). Scatter plots for PLZF expression within the indicated cells are shown (right). **(B)** Representative flow cytometric dot plots showing the expression of PLZF within the NKG2A/C/E^+^ CD4^+^ and NKG2A/C/E^+^ DN cells from Jα18^−/−^MHCII^−/−^ mice. **(C)** Splenocytes depleted of cells positive for B220, CD11b, CD11c and CD8α were stimulated with PMA and ionomycin, and thereafter stained for surface markers and intracellular cytokines. Representative flow cytometric contour plots showing the expression of indicated cytokines within B220^−^CD8^−^TCRβ^+^CD4^+^NKG2D^+^ cells. Results are expressed as mean ± SD. Each dot represents one experiment with splenocytes pooled from 2 mice.
